# Feasibility and Links Between Emotions, Physical States, and Eating Behavior in Patients After Metabolic Bariatric Surgery: Experience Sampling Study

**DOI:** 10.2196/60486

**Published:** 2025-03-05

**Authors:** Ellen A M Kuipers, Josien G Timmerman, Marc J van Det, Miriam M R Vollenbroek-Hutten

**Affiliations:** 1 Department of Surgery Hospital Group Twente Almelo The Netherlands; 2 Biomedical Signals and Systems Faculty of Electrical Engineering, Mathematics and Computer Science University of Twente Enschede The Netherlands; 3 ZGT Academy Hospital Group Twente Almelo The Netherlands; 4 Board of Directors Medisch Spectrum Twente Enschede The Netherlands

**Keywords:** feasibility, experience sampling methodology, metabolic bariatric surgery, eating behavior, positive and negative affect, physical states, contextual factors, mobile phone

## Abstract

**Background:**

Lifestyle modification is essential to achieve and maintain successful outcomes after metabolic bariatric surgery (MBS). Emotions, physical states, and contextual factors are considered important determinants of maladaptive eating behavior, emphasizing their significance in understanding and addressing weight management. In this context, experience sampling methodology (ESM) offers promise for measuring lifestyle and behavior in the patient’s natural environment. Nevertheless, there is limited research on its feasibility and association among emotions and problematic eating behavior within the population after MBS.

**Objective:**

This study aimed to examine the feasibility of ESM in the population after MBS regarding emotions, physical states, contextual factors, and problematic eating behavior, and to explore the temporal association among these variables.

**Methods:**

An experience sampling study was conducted in which participants rated their current affect (positive and negative), physical states (disgust, boredom, fatigue, and hunger), contextual factors (where, with whom, and doing what), and problematic eating behavior (ie, grazing, dietary relapse, craving, and binge eating) via smartphone-based ESM questionnaires at 6 semirandom times daily for 14 consecutive days. Feasibility was operationalized as the study’s participation rate and completion rate, compliance in answering ESM questionnaires, and response rates per day. At the end of the study period, patients reflected on the feasibility of ESM in semistructured interviews. Generalized estimation equations were conducted to examine the temporal association between emotions, physical states, contextual factors, and problematic eating behavior.

**Results:**

In total, 25 out of 242 participants consented to participate, resulting in a study participation rate of 10.3%. The completion rate was 83%. Overall compliance was 57.4% (1072/1868), varying from 13% (11/84) to 89% (75/84) per participant. Total response rates per day decreased from 65% (90/138) to 52% (67/130) over the 14-day study period. According to the interviews, ESM was considered feasible and of added value. Temporal associations were found for hunger and craving (odds ratio 1.04, 95% CI 1.00-1.07; *P*=.03), and for positive affect and grazing (odds ratio 1.61, 95% CI 1.03-2.51; *P*=.04).

**Conclusions:**

In this exploratory study, patients after MBS were not amenable to participate. Only a small number of patients were willing to participate. However, those who participated found it feasible and expressed satisfaction with it. Temporal associations were identified between hunger and craving, as well as between positive affect and grazing. However, no clear patterns were observed among emotions, physical states, context, and problematic eating behaviors.

## Introduction

Metabolic bariatric surgery (MBS) is the most effective long-term strategy in the management of severe obesity and its related comorbidities [1]. Lifestyle modification, including nutritional compliance and physical activity, is essential to achieve and maintain optimal weight loss results and to improve obesity-related comorbidities after MBS [2-4]. Poor dietary adherence is significantly associated with recurrent weight gain after MBS [1,5,6]. Furthermore, maladaptive eating behaviors may cause nutritional noncompliance with the inability to remain consequently adherent to an intended diet (ie, dietary relapse) and thereby negatively impact postsurgical weight loss outcomes [7-10]. Most theoretical models of eating disorders posit that negative emotions and numerous physical states (ie, hunger and fatigue) contribute to the development and maintenance of maladaptive eating behaviors [11-17]. However, little is known whether these theoretical constructs apply to individuals who have undergone MBS. To our best knowledge, only 1 study has explored the relationship between negative affect and loss of control (LOC) eating in patients who underwent MBS, finding that higher momentary negative affect predicted more severe LOC eating both before and after MBS [18]. Since this study focused on 1 aspect of disordered eating behavior, it is important to enhance our understanding of how emotions, physical states, and other maladaptive eating behaviors are interconnected in this specific population. This is particularly important given the high prevalence of problematic eating behaviors in the population with obesity, including grazing, food cravings, and binge eating [7,19-22]. Grazing, defined as unplanned and repetitive eating of small amounts of food between meals and snacks [23], is reported to occur in up to 26% of patients seeking MBS [7]. Postoperatively, the prevalence is even higher, with rates reported as high as 47% [8]. Food cravings, characterized by an intense desire to consume specific foods, are experienced by nearly all patients after MBS, with only about 10% reporting no cravings [24]. Binge eating involves the consumption of excessive amounts of food within a discrete period of time, accompanied by an LOC feeling during the binge eating episode [9]. Binge eating, which is present in 24% of patients before MBS [25], is strongly associated with obesity and negatively impacts weight outcomes after MBS [8].

Since lifestyle and lifestyle modification after MBS takes place primarily outside of medical visits and clinical settings, there is an urgent need to have ecologically valid methods that capture daily life to better understand and change behaviors. Experience sampling methodology (ESM), also known as ecological momentary assessment, addresses this need by collecting real-time data on individuals’ behavior, thoughts, emotions, and environmental contexts as they go about their daily lives [26]. Unlike retrospective questionnaires, ESM prompts participants at random or scheduled times throughout the day to record their current state or activities. This approach provides a more accurate representation of behavior within the individual’s natural environment, capturing their typical routines in daily life [27], while traditional self-reports may miss crucial details about how and why a participant’s experiences or behaviors change over time and context.

In the past years, an increasing number of experience sampling studies have been conducted in various health domains, representing a wide range of diseases and populations [17,28-37]. However, the use of ESM to evaluate (disordered) eating behaviors following MBS remains limited [18,38,39]. Previous research has focused on specific aspects, such as 1 type of problematic eating behavior [18], eating practices that do not align with postoperative dietary or eating behavior recommendations (eg, drinking liquids with meals and not starting meals with protein) [39], or elements of eating regulation (eg, dietary restraint and eating in the absence of hunger) [38]. Our goal is to conduct a more comprehensive assessment by examining a broader range of problematic eating behaviors and their influencing factors—an important objective given the high prevalence of disordered eating behavior in the population after MBS [20], as outlined earlier in this section.

The feasibility of ESM has been previously evaluated in various populations, including its use to monitor dietary intake and physical activity among Dutch vocational education students [28], to study depressive symptoms among caregivers of patients with cancer [31], and to examine daily life experiences and activities in patients with acute coronary syndrome [33]. These studies show varying results regarding compliance, participation rates, and completion rates, indicating that feasibility for ESM differs between populations. However, the feasibility of ESM has scarcely been studied in the population that has undergone or is undergoing MBS. To our knowledge, only 1 experience sampling feasibility study was conducted in adults undergoing MBS, focusing on the use of a smartphone app for lifestyle education before surgery [40]. This study reported high satisfaction and perceived helpfulness, but the study was carried out in patients before MBS. After MBS, patients are dealing with physical and mental changes, along with the need to modify their behavior. This may impact the feasibility of ESM, as patients are likely to prioritize managing these challenges over using an experience sampling app for monitoring. Although some studies have used ESM to evaluate (disordered) eating behaviors after MBS [18,38,39,41], they did not specifically focus on evaluating feasibility. While these studies reported compliance, they did not assess other feasibility metrics. Notably, participants in these studies received monetary compensation [38,39,41], which may have positively influenced participation and compliance, potentially affecting feasibility outcomes [42]. This raises concerns about the generalizability of these findings to the real-world clinical setting.

Therefore, the primary aim of this exploratory study is to establish the feasibility of ESM among patients shortly after MBS, focusing on maladaptive eating behaviors, emotions, physical states, and context. The secondary aim is to explore associations between emotions, physical states, context, and maladaptive eating behaviors in the population after MBS using experience sampling measurements.

## Methods

### Study Design and Participants

A prospective observational cohort study was performed at the obesity center of Hospital Group Twente (ZGT) Almelo/Hengelo, the Netherlands. All patients who underwent MBS at ZGT during the inclusion period (from February 2022 to July 2022) were considered for participation in this study. We included patients who had recently undergone MBS, as lifestyle modification mostly occurs after surgery, making this period particularly relevant for monitoring to enable timely intervention. The inclusion and exclusion criteria are detailed in Textbox 1.

**Inclusion and exclusion criteria.**

**Inclusion criteria**

- Internet access at home
- Good comprehension of the Dutch language

**Exclusion criteria**

- No possession of a smartphone that supports the experience sampling methodology app
- Participation in an intervention program or behavioral treatment that is not included in the regular care pathway after metabolic bariatric surgery
- People who worked on night shifts (as it is impossible to complete the experience sampling questionnaires during the day)

### Study Procedure

#### Recruitment

Patients were initially recruited through flyers sent by post and by email 5 weeks after surgery. Due to a minimal response, the recruitment strategy was expanded; patients were approached following a postoperative group meeting or patients were contacted by telephone. Recruitment proceeded as follows:

1. Via flyer by post or email: Participant information was provided to the patients by scanning the QR code on the flyer or clicking on the link. If the patient was interested in participating after reading the information, the patient could send an email to the first author (EAMK). Later, individuals were contacted via telephone by the first author (EAMK) or a student (Charlotte van den Berg) to provide verbal information about the study.
2. During a postoperative group meeting: A student (Charlotte van den Berg) provided verbal and written information about the study. If interested, patients shared their contact details for inclusion. Furthermore, a subset of these patients was contacted by phone to encourage participation.
3. By telephone: Postoperative patients were contacted by telephone by a student (Charlotte van den Berg) or by the first author (EAMK) to inform patients about the study and motivate them to participate.

After inclusion, participants received verbal instructions on installing and using the ESM app. A user manual for the ESM app was sent via email. The participants used their own email addresses to create an account in the ESM app Ethica Data. After entering a unique registration code to join the study, participants were briefed on the study, and at this point, participants signed informed consent in the Ethica Data app. Patients who consented were asked if they would like to participate in an interview at the end of the study period to discuss the feasibility of the ESM app.

#### Questionnaires and Interviews

Participants were instructed to answer ESM questionnaires for 14 consecutive days, to capture both weekdays and weekends. The app was programmed to send auditory and visual notifications at 6 semirandom times throughout the day (1 prompt every 135 min, between 8:00 AM and 9:30 PM), signaling the availability of a questionnaire (signal contingent). At each prompt, participants answered questions regarding current positive and negative affect, physical states, context, and problematic eating behavior. A delayed response triggered a 1-time reminder after 5 minutes, and each questionnaire expired after 15 minutes to minimize recall bias. Participants were asked to carry their smartphones throughout the day and continue their daily lives as usual. Participants who consented to the interview were contacted by phone to discuss the feasibility of the ESM app.

### Data Collection

#### Baseline Measures

Demographics and surgical data were obtained from electronic patient records. Collected patient characteristics included age; gender; type of surgery; time since surgery; and obesity-related comorbidities, such as hypertension, diabetes mellitus, dyslipidemia, osteoarthrosis, gastroesophageal reflux disease, and obstructive sleep apnea syndrome. At the start of the study, each participant was asked to enter their current weight and height into the Ethica Data app.

#### Feasibility

Feasibility was operationalized as participation rate, completion rate, compliance, and response rate per day.

- The participation rate was calculated as the percentage of approached patients willing to participate in the study.
- The completion rate represented the percentage of participants who filled out at least 1 ESM questionnaire on the 14th day of the study period.
- Compliance with experience sampling was calculated as the percentage of answered ESM questionnaires out of all issued ESM questionnaires.
- The response rate per day was the percentage of ESM questionnaires answered per day, calculated for each day of the study period.

At the end of the 2-week period, participants were invited to participate in a semistructured interview to share their experiences. The interviews were conducted by the first author (EAMK) and a master’s student in health science (Charlotte van den Berg) via telephone in November and December 2022. The interviews, guided by a semistructured interview guide (Multimedia Appendix 1), lasted between 15 and 30 minutes. This guide covered the feasibility themes to be discussed, including user-friendliness, technical performance, and content, along with optional questions. With participants’ permission, the interviews were audio-recorded, transcribed verbatim, and anonymized.

#### ESM Measures

ESM data were collected using the Ethica Data app. All ESM questions were provided in the patients’ native language (Dutch). ESM data included the following items:

1. Emotion: Participants rated their feelings as “angry or annoyed,” “anxious or scared,” “relaxed or calm,” “cheerful or happy,” “sad or gloomy,” and “tensed or stressed” [43,44]. These items reflect both positive and negative affect and were scored on a visual analog scale from 0 (not at all or very bad) to 10 (extremely or very good).
2. Physical states: Disgust, boredom, fatigue, and hunger were rated. The first ESM questionnaire in the morning also asked participants to rate their sleep quality. Physical state items were scored on a visual analog scale from 0 to 10.
3. Context and activities: Questions about the context (where, with whom) and activities (doing what) had multiple-choice response options.
4. Problematic eating behavior: Participants answered 4 questions about problematic eating behavior. The questions and answer options are presented in Table 1.

**Table 1:** Experience sampling questions about problematic eating behavior and their answer options.

Behavior	Question	Answer option
Craving	“Have you had a craving for a specific food in the past 30 minutes? If yes, how strong was this desire?”	VAS^a^: 0-10
Dietary relapse	“In the past 60 minutes, did you eat and/or drink anything that is likely to negatively impact your weight?”	Yes or no
Binge eating	“Have you eaten unusually large amounts of unhealthy food in the past 60 minutes?”	Yes or no
Grazing	“In the past 60 minutes, have you eaten unplanned and repetitive small amounts of food outside of planned meals and snacks?”	Yes or no

^a^VAS: visual analog scale.

### Statistical Analysis

Statistical analysis was performed using SPSS (version 24; IBM Corp). Patient characteristics were expressed as means with SDs for parametric continuous data or frequencies and percentages for categorical data. For nonparametric continuous data, median and IQR were reported. Feasibility metrics were expressed as percentages.

To investigate the relationship between emotions, physical states, context, and problematic eating behaviors, generalized estimation equations (GEEs) were used. GEEs are an extension of generalized linear models designed to account for the correlation between repeated measures within participants, as data collected through ESM involve multiple observations per individual [45]. This makes GEEs the appropriate choice for our analysis, as they allow us to model repeated measures data while adjusting for within-subject correlations without assuming that these correlations are the same across all participants.

We used a negative binomial distribution with a binary logistic link function, as our dependent variables (eg, dietary relapse and grazing) were dichotomous (yes or no) and did not follow a normal distribution. The negative binomial distribution is particularly suitable for overdispersed count data, which are often encountered in studies involving behavioral data. An exchangeable correlation structure was selected, assuming a constant correlation between repeated measures within individuals over time. While centering is customary in ESM studies, in this study, not centering the variables aligns better with the interpretability of the results. Retaining the raw values ensures that the intercept is directly relatable to real-world states (eg, no negative or positive emotion), which facilitates reader comprehension without altering the estimated effects.

Momentary assessments (level 1) were nested within participants (level 2). Momentary levels of positive affect were calculated by averaging the scores of the “relaxed or calm” and “cheerful or happy” items, while momentary levels of negative affect were calculated by averaging the “angry or annoyed”, “anxious or scared”, “sad or gloomy” and “tensed or stressed” items. Contextual factors were recoded as dichotomous variables such as “being at home’ (yes or no), “being alone” (yes or no), and “relaxing” (yes or no), where “relaxing” encompassed resting or doing nothing. Due to nonnormality, craving was dichotomized (yes or no).

To account for potential temporal dependencies, we lagged the independent variables (emotions, physical states, and contextual factors) by 1 time point, meaning that the predictors at time *t*–1 were used to examine their influence on problematic eating behavior at time *t*. The first assessment of each day was excluded from the analysis to avoid carry-over effects from the previous day.

A minimum response rate of 33% for ESM data was applied, which is the generally accepted threshold for ESM studies [46]. Univariate GEE analyses were conducted for each predictor variable, and given the exploratory nature of the study, we did not adjust for multiple comparisons. The significance level was set at *P*<.05.

### Thematic Analysis

Using the software program ATLAS.ti (version 22; Lumivero), the interview transcripts were reviewed and analyzed thematically by the first author. Initially, each transcript was read multiple times without conducting a thematic analysis. Next, comments related to feasibility issues were extracted from the transcripts without interpretation or categorization. These feasibility issues were then classified deductively according to the 7 categories of the eHealth Usability Benchmarking Instrument (HUBBI), namely, basic system performance, task-technology fit, interface design, navigation and structure, information and terminology, guidance and support, and satisfaction [47]. Finally, transcripts were reread to ensure that all relevant codes were identified.

### Ethical Considerations

The study protocol was reviewed and approved by the local medical ethics committee (local registration 2020-48), and all patients provided informed consent. Data collected via the Ethica Data app were securely stored on Ethica Data’s servers. Ethica Data employs 2 separate databases located on 2 different servers. Participant registration data (ie, name and email) are stored on one server, while study data (ie, questionnaire responses) are stored on another. Study data are stored in a coded format and cannot be traced back to individual participants. The study data stored on Ethica Data’s server were accessible only to researchers with a password-protected account and could be imported by the researchers to ZGT’s server. The dataset contains only participant numbers, and the combination of participant numbers and email addresses was securely stored in a password-protected file within the research department. Only authorized persons had access to these data. Audio recordings of interviews were used to generate deidentified transcripts, after which the recordings were immediately destroyed. Both the audio recordings and transcripts were securely stored on a protected hard drive. Participants with a minimum response rate of 80% on the ESM questionnaires had the chance to win a €50 voucher (US $52.32), which was randomly awarded to 1 participant. No additional monetary compensation was provided.

## Results

### Participants

**Figure 1:**
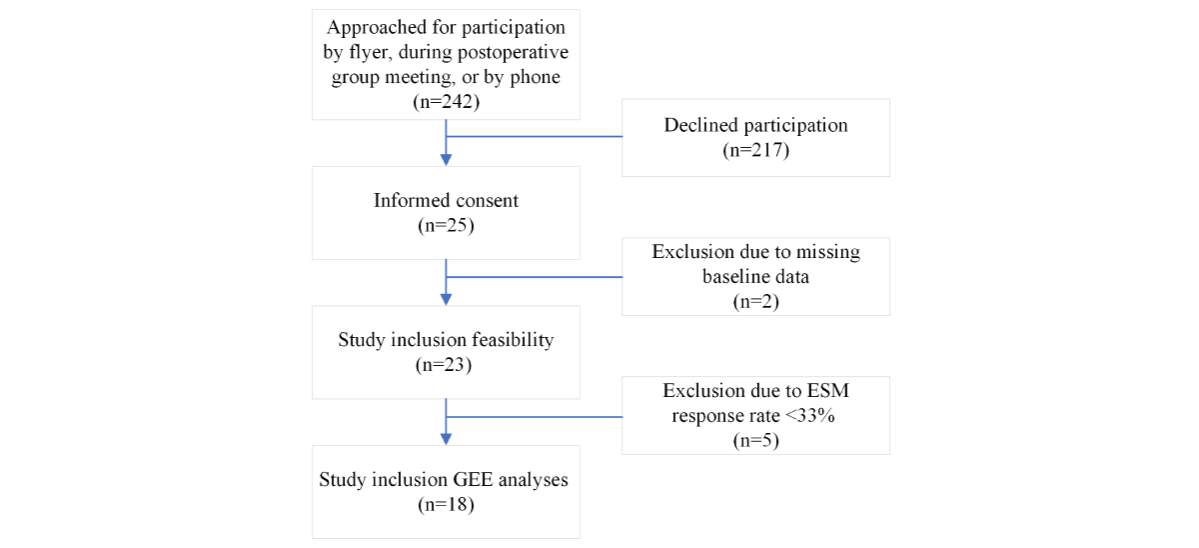
Study flowchart of patients approached for participation between February and July 2022. ESM: experience sampling methodology; GEE: generalized estimation equation.

From February to July 2022, a total of 242 patients were informed about the study. Out of the 242 patients who were approached to participate in the ESM study, 25 patients consented to participate, resulting in a participation rate of 10.3%. Figure 1 presents the study flowchart.

Two participants were excluded from analyses due to missing baseline data. The remaining 23 participants were included to answer the primary aim of the study. Although 2 participants withdrew due to a high perceived burden, they were included in the feasibility analysis. For the secondary aim of this study, an additional 5 participants were excluded due to a response rate of less than 33% for all ESM questionnaires. Thus, a total of 18 participants were included in this analysis.

### Demographics

The 23 participants had a mean age of 47 (SD 11) years, and 17 (74%) were female. The median time since surgery was 36 (IQR 31-88) days. At inclusion, median BMI was 36 (IQR 33-39) kg/m^2^. The most performed bariatric procedure was one-anastomosis gastric bypass (n=16, 70%). The most prevalent obesity-related comorbidities were dyslipidemia (n=21, 91%) and osteoarthrosis (n=13, 57%). Descriptive statistics of the participants are shown in Table 2.

**Table 2:** Participant characteristics for the total study population, as well as for those with a response rate ≥33% and <33% on the experience sampling questionnaires.

Participant characteristic			Total (n=23)		Response rate ≥33% (n=18)		Response rate <33% (n=5)
Age (years), mean (SD)			47 (11)		49 (12)		42 (9)
**Sex, n (%)**							
	Female	17 (74)		13 (72)		4 (80)	
	Male	6 (26)		5 (28)		1 (20)	
BMI (kg/m^2^), median (IQR)			36 (33-39)		36 (33-38)		39 (32-42)
Days between surgery and study start, median (IQR)			36 (31-88)		34 (28-79)		88 (60-103)
**Comorbidities, n (%)**							
	T2DM^a^	3 (13)		2 (11)		1 (20)	
	Hypertension	10 (44)		9 (50)		1 (20)	
	OSAS^b^	8 (35)		7 (39)		1 (20)	
	Osteoarthrosis	13 (57)		10 (56)		3 (60)	
	GERD^c^	10 (44)		8 (44)		2 (40)	
	Dyslipidemia	21 (91)		17 (94)		4 (80)	
**Type of surgery, n (%)**							
	SG^d^	1 (4)		0 (0)		1 (20)	
	RYGB^e^	3 (13)		3 (17)		0 (0)	
	OAGB^f^	16 (70)		13 (72)		3 (60)	
	OAGB+correction HH^g^	2 (9)		1 (6)		1 (20)	
	OAGB+cholecystectomy	1 (4)		1 (6)		0 (0)	

^a^T2DM: type 2 diabetes mellitus.

^b^OSAS: obstructive sleep apnea syndrome.

^c^GERD: gastroesophageal reflux disease.

^d^SG: sleeve gastrectomy.

^e^RYGB: Roux-en-Y gastric bypass.

^f^OAGB: one-anastomosis gastric bypass.

^g^HH: Hiatal hernia.

### Feasibility

#### Overview

The compliance per participant ranged from 13% (11/84 questionnaires completed) to 89% (75/84 questionnaires completed). Overall, participants responded to 1072 out of 1868 prompts, resulting in an overall compliance of 57.4%. The compliance in answering experience sampling questionnaires per participant is presented in Multimedia Appendix 2.

**Figure 2:**
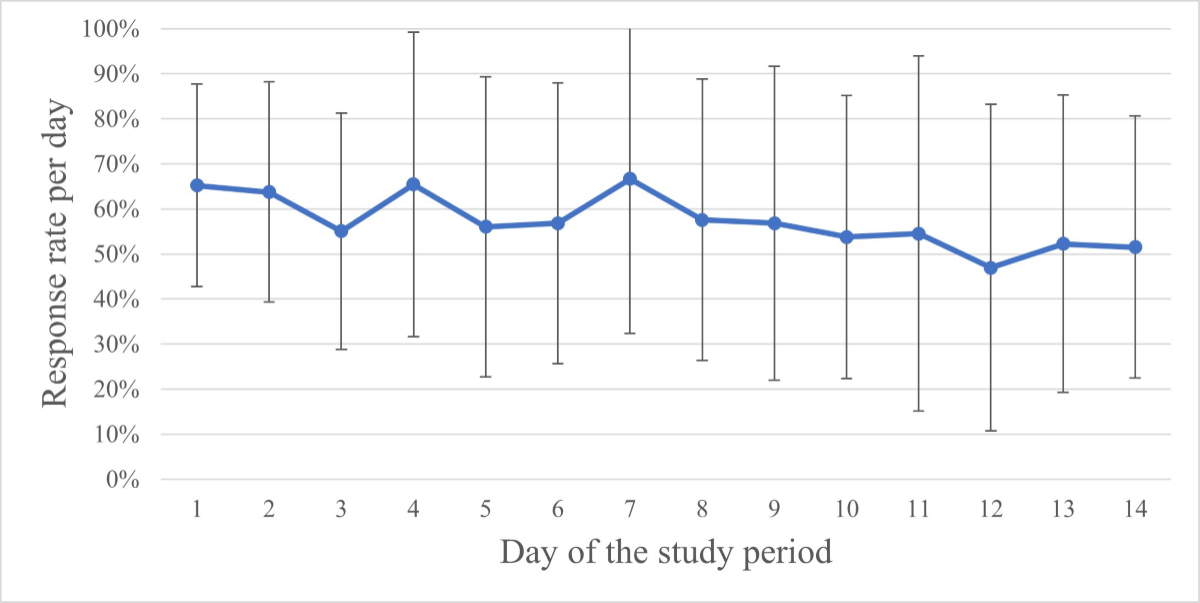
Mean response rates per day and SD for the total study population (n=23) over 14 days of study participation.

The mean number of answered ESM questionnaires was 47 (SD 21). Total response rates were calculated from day 1 to day 14, as illustrated in Figure 2. Over time, the total response rate declined by 14%. The study completion rate was 83% (19/23).

#### Semistructured Interviews

##### Overview

In total, 10 patients participated in the semistructured interviews, of whom 7 (70%) were female. The mean age was 46 (range 28-64) years, and the mean BMI was 38 (SD 6) kg/m^2^. The median time between surgery and participation in the ESM study was 34 (IQR 28-87) days.

The categories that were indicated in the interviews, based on the HUBBI, are basic system performance, task-technology fit, information and terminology, guidance and support, and satisfaction [47].

##### Basis System Performance

The participants reported that the app was easy to use and straightforward to install on their mobile phones, with no technical issues during installation. When asked about technical performance in general, 1 participant reported repeated technical problems that prevented her from completing several questionnaires. In addition, she reported not receiving auditory notifications and sometimes inconsistent visual notifications.

##### Task-Technology Fit

Most participants reported they could not complete all questionnaires within the 15-minute time frame due to work or travel commitments. Participants perceived the number of questions per questionnaire as appropriate and found them easy to answer since the questionnaires were nearly identical. The daily number of questionnaires per day was also considered manageable, with 1 participant stating:

Not too much, not too little [the number of questionnaires].

Participant 6

Another participant felt that 6 questionnaires per day was the maximum, saying:

Otherwise, you spend so much time working on it [the questionnaires], I don’t think that’s healthy.

Participant 7

Furthermore, 3 participants shared their views on the 2-week survey duration, and 2 of them indicated that a longer duration would have been acceptable, with 1 noting:

If it’s not going well immediately [after MBS], it [filling in the questionnaires] can provide insights, because it’s quite astonishing how you change after bariatric surgery.

Participant 6

In addition, 7 participants indicated that using the app encouraged them to reflect on their behavior, providing insights and increasing awareness. One participant found the questions negatively framed, which led to his reluctance to use the app, ultimately causing him to withdraw from the study. Another participant felt she was doing very well at that moment. Therefore, she experienced no additional support by using the app.

##### Information and Terminology

The participants found the questions clear, though 5 participants noted that the questions were repetitive. One participant commented:

Maybe I expected different questions, as not much has changed in such a short time [time since previous questionnaire].

Participant 10

Another participant suggested occasionally changing the order of the questions:

This way, you have to think carefully about which question to answer.

Participant 2

Furthermore, 2 participants would have preferred open questions as well:

Imagine you experience a certain emotion or that you ate something ‘wrong’, what was that about? If you could explain a little more, it would provide greater depth.

Participant 10

As mentioned above, 1 participant indicated that the questions were negatively framed, feeling that the negative aspects overshadowed questions about positive emotions.

##### Guidance and Support

The participants were not bothered about lacking insight into their own answers, understanding it was for scientific research. As one stated:

It is especially important for you as researchers, I don’t necessarily have to see it myself [the answers].

Participant 3

However, 5 participants felt that having access to their answers could be valuable. One of them mentioned:

It might be interesting to see if there were any changes or patterns.

Participant 10

Another remarked:

On one hand, I see the benefit, on the other, it could raise many questions. Ultimately, if things aren’t going well, you’re the one who must seek help.

Participant 6

Overall, participants were positive about using the app in bariatric care, as it could help individuals become more aware of their actions:

I can imagine that some people have had surgery and then feel a little lost. Of course, you have group meetings with the dietician, but the application allows for personal reflection in a quieter moment.

Participant 5

In total, 4 participants indicated that using the app before MBS could be interesting:

Are people aware of their emotions and their eating behavior before surgery?

Participant 2

Participants expressed mixed opinions on how an app should be integrated into follow-up care after MBS. One suggested that questions about changes in eating patterns, the creeping in of old habits, physical activity, and mental well-being may be relevant 1 or 2 years after MBS. Another participant expressed interest in questions about nutrition and related uncertainties. One participant preferred an app where she could ask questions directly to a health care professional. Participants recognized the need to tailor the app to their specific stage after surgery. While most participants preferred using the app in the short term (within a few months) after MBS, 1 participant preferred using the app on a longer-term (at least 6 months) after MBS. He indicated that, in the short term after surgery, he would have benefited more from an app that provided meal or snack suggestions, and calculated protein intake based on those suggestions.

##### Satisfaction

In general, participants were positive about the app, finding it helpful in the follow-up after MBS. Most participants experienced personal benefits from using the app, noting that it encouraged more conscious eating by prompting questions at random times:

What did I actually eat or drink and how do I feel about that?

Participant 2

One participant experienced a personal disadvantage by using the app:

I remember thinking that if I continue this [the questionnaires], it will even make me depressed. I don’t think that’s the intention.

Participant 9

For improvement, participants suggested extending the time a questionnaire remains open and adding the question:

What do all these questions do to you?

Participant 2

If the answer is negative, it was recommended that the app alert the health care professional:

I think you should contact this person, because he can’t figure it out on his own.

Participant 2

### Association Between Positive and Negative Affect, Contextual Factors, and Problematic Eating Behavior

#### Frequencies and Percentages of Problematic Eating Behaviors

Frequencies and percentages of the different types of problematic eating behavior are illustrated in Multimedia Appendix 3.

- Dietary relapse: In total, 6 participants reported no dietary relapse over 14 days. The remaining participants reported a total of 45 dietary relapses, with individual counts ranging from 1 to 8.
- Grazing: Grazing occurred in total 13 times among 7 participants (range per participant: 1-3), while 11 participants reported no grazing during the study period.
- Binge eating: A total of 7 binge eating episodes were reported by 3 participants (range per participant: 2-3), while 15 participants reported no binge eating episodes during the study period.
- Craving: Craving was absent (score 0) in 65% (643/994) of all completed questionnaires. In addition, 3 participants consistently reported no craving throughout the study, while the remaining 15 participants experienced a total of 351 craving episodes (range per participant: 3-53).

#### GEE Analyses

Data from the ESM questionnaires were nested within 18 individuals. The number of observations per predictor or outcome variable ranged from 987 to 1005. After lagging each predictor and setting the first prompt of each day to missing, the number of observations available for the GEE analyses ranged from 594 to 605. Table 3 summarizes the GEE results, showing the temporal associations between lagged predictors (ie, positive and negative affect, physical states, and contextual factors) and problematic eating behaviors (craving, binge eating, dietary relapse, and grazing). Significant associations are described below, while nonsignificant trends are noted for exploratory purposes.

- Craving: Hunger was significantly associated with craving (OR 1.04, 95% CI 1.00-1.07; *P*=.03).
- Binge eating: The odds of binge eating increased by 1.61 times with each unit increase in negative affect (95% CI 0.77-3.34; *P*=.21) and decreased by 0.51 times for each unit increase in disgust (95% CI 0.20-1.28; *P*=.15). When at home, the odds of binge eating decreased by 0.27 times compared with not being at home (95% CI 0.03-2.67; *P*=.26).
- Dietary relapse: The odds of dietary relapse decreased by 0.40 times for being at home (95% CI 0.14-1.17; *P*=.09) and by 0.67 times for each unit increase in boredom (95% CI 0.40-1.14; *P*=.14).
- Grazing: A significant temporal association was found for positive affect and grazing, with the odds of grazing increasing by 1.61 times for each unit increase in positive affect (95% CI 1.03-2.51; *P*=.04). The odds of grazing decreased by 0.68 times with each unit increase in negative affect (95% CI 0.40-1.15; *P*=.15). When at home, the odds of grazing decreased by 0.16 times compared with not being at home (95% CI 0.02-1.07), with this result approaching statistical significance (*P*=.06).

**Table 3:** Generalized estimation equation analyses showing the temporal associations between lagged predictors (positive and negative affect, physical states, and contextual factors) and subsequent problematic eating behaviors (craving, binge eating, dietary relapse, and grazing) in patients shortly after metabolic bariatric surgery.

Eating behavior and predictor		Odds ratio (95% CI)	*P* value
**Craving**			
	Negative affect	1.06 (0.79-1.42)	.69
	Positive affect	1.02 (0.91-1.15)	.70
	Boredom	1.03 (0.89-1.20)	.70
	Disgust	1.01 (0.82-1.25)	.91
	Fatigue	1.07 (0.96-1.18)	.21
	Hunger	1.04 (1.00-1.07)	.03
	Being alone	1.01 (0.87-1.17)	.92
	Being at home	1.15 (0.93-1.42)	.21
	Relaxing	0.93 (0.78-1.09)	.36
**Binge eating**			
	Negative affect	1.61 (0.77-3.34)	.21
	Positive affect	0.95 (0.53-1.69)	.86
	Boredom	0.93 (0.57-1.50)	.76
	Disgust	0.51 (0.20-1.28)	.15
	Fatigue	1.10 (0.81-1.50)	.55
	Hunger	1.00 (0.68-1.48)	≥.99
	Being alone	0.80 (0.11-5.81)	.83
	Being at home	0.27 (0.03-2.67)	.26
	Relaxing	0.80 (0.17-3.91)	.79
**Dietary relapse**			
	Negative affect	0.84 (0.46-1.52)	.56
	Positive affect	1.15 (0.91-1.46)	.23
	Boredom	0.67 (0.40-1.14)	.14
	Disgust	0.89 (0.59-1.36)	.59
	Fatigue	1.14 (0.90-1.45)	.28
	Hunger	0.89 (0.72-1.10)	.28
	Being alone	0.82 (0.35-1.91)	.65
	Being at home	0.40 (0.14-1.17)	.09
	Relaxing	0.97 (0.30-3.20)	.97
**Grazing**			
	Negative affect	0.68 (0.40-1.15)	.15
	Positive affect	1.61 (1.03-2.51)	.04
	Boredom	0.81 (0.43-1.55)	.53
	Disgust	0.88 (0.57-1.38)	.59
	Fatigue	1.23 (1.00-1.51)	.06
	Hunger	0.77 (0.55-1.09)	.15
	Being alone	1.14 (0.33-3.94)	.83
	Being at home	0.16 (0.02-1.07)	.06
	Relaxing	0.90 (0.14-5.97)	.91

## Discussion

### Principal Results

This exploratory study describes the feasibility of ESM in patients after MBS, focusing on negative and positive affect, physical states, contextual factors, and problematic eating behaviors. The results indicate that patients shortly after MBS may be less inclined to participate in experience sampling studies. Nonetheless, actual usage was considered feasible, given the moderate compliance and high completion rate upon patient participation. In addition, results from the semistructured interviews indicated that the ESM app used in this study showed to be usable and of added value from the patient’s perspective. This indicates that patients who chose to participate were motivated to complete the ESM questionnaires. Concerning the second research aim, temporal associations were found, but only between hunger and craving, and between positive affect and grazing.

### Comparison With Previous Work

Our study faced a low participation rate of 10.3%. Several participants who were approached face-to-face and declined participation, mentioned the high expected burden, or indicated that study participation would likely not fit into their daily life or work schedule. Ratcliff et al [48] performed an experience sampling feasibility study to assess the adherence to postoperative diet and activity patterns in adolescents who underwent MBS. Their participation rate was 62%, which is remarkably higher than ours. However, they included participants at least 12 months after MBS, while we approached patients eligible for participation a few weeks after MBS. This timing difference may have influenced the willingness to participate, considering the overwhelming changes patients are dealing with in the first weeks after surgery. Furthermore, in the study by Ratcliff et al [48], participants completed only 3 daily phone diaries (a form of ESM) instead of 14 days of measurements. As we were interested in the feasibility of ESM, we opted for an intensive sampling strategy to gain insight into the daily response rates throughout the study period. This may have negatively affected our participation rate because of the high expected burden shortly following MBS. In addition, to obtain more realistic feasibility metrics, patients were not incentivized with monetary remuneration, except for 1 voucher randomly awarded among participants with a minimum response rate of 80%. This approach offers insights into the effectiveness of ESM in clinical practice without the use of financial incentives but might have further contributed to a low participation rate. Finally, we recruited patients through flyers sent by post and email, as well as during group meetings. An individualized or personalized recruitment strategy could potentially increase participation rates.

Our compliance rate was 56%, while previous experience sampling studies regarding affect and problematic eating behaviors showed higher compliance rates ranging from 62% to 81% [17,44,49,50]. Interestingly, compliance rates were higher (62%) in the studies by Stevenson et al [50] and Boh et al [44] (80% for participants with healthy weight and 81% for participants who were overweight), despite both studies administering more daily ESM questionnaires over a similar study duration compared with ours. First, differences in study populations may account for the discrepancy in compliance rates. For instance, Boh et al [44] included participants who were both healthy-weight and overweight, but none of these participants had recently undergone MBS. MBS entails associated changes and challenges, particularly in the short term following surgery. This might have played a role in the lower compliance rates observed in our study. Participants in the study by Stevenson et al [50] were younger compared with our participants (age 24 years versus 47 years in our study). This could potentially explain their higher compliance rates, as mobile apps for self-management are known to be used less frequently by older adults compared with younger individuals [51,52]. Second, to minimize recall bias, we restricted the time window for participants to respond to ESM questionnaires to 15 minutes. Several participants mentioned in the semistructured interviews that this short-time window hindered their ability to respond, possibly contributing to our lower compliance rate. However, other ESM studies with longer response windows have reported compliance rates that are either lower or similar to ours [28,31], whereas others with similar time windows achieved higher compliance rates [33]. This indicates that extending the time window or increasing reminder frequency, is unlikely to positively impact compliance within our study population.

This study is one of the few to assess problematic eating behaviors using ESM in the population after MBS [18,38,39]. While different methods exist for assessing problematic eating behaviors, we used terminology consistent with previous research in populations with overweight or obesity [53,54]. Our study identified a significant temporal association between hunger and craving (*P*=.03). However, an OR close to 1 (OR 1.04) indicates a minimal effect of hunger on craving. Previous studies also found that food cravings closely follow hunger [55,56]. Given that food craving is defined as an intense desire to consume a specific food, and hunger as an urgent need for food or a specific nutrient, it is unsurprising that both constructs are closely intertwined. This overlap might create difficulties for participants to differentiate between both constructs and therefore, an association between hunger and craving could have arisen. Our results also revealed a significant temporal association between positive affect and grazing (OR 1.61, 95% CI 1.03-2.51; *P*=.04), where grazing behaviors increased with higher levels of positive affect. As grazing behaviors were not specifically assessed in previous studies, direct comparisons with existing literature are not possible. However, practical implications arise from the identified associations. For example, recognizing a positive mood might help patients in acknowledging the potential risk of grazing behavior.

Overall, our study yielded inconclusive results regarding the association between negative emotions, positive emotions, and problematic eating behaviors, as no clear patterns emerged from the GEE analyses. ORs regarding problematic eating behaviors for both positive and negative emotions (including boredom and disgust) were notably ambiguous. This is unexpected, as most theoretical models of eating disorders propose that negative emotions trigger episodes of problematic eating behavior [13]. It is likely that the impact of MBS, which encompasses both mental and physical adjustments, might have influenced the inconsistencies in positive and negative affect on problematic eating behaviors in our study. Future studies focusing on the longer term after MBS are needed to assess the association between both positive and negative affect and problematic eating behaviors. Furthermore, participants in our study exhibited fewer episodes of problematic eating behaviors compared with existing literature [57], resulting in a low number of events, which likely impacted the robustness of our analyses. Consequently, the limited frequency of problematic eating episodes observed in our sample, combined with potential influences of post-MBS adaptations, may have contributed to the inconclusive findings regarding the role of emotions in problematic eating behaviors.

### Study Strengths and Limitations

As we were interested in the feasibility of ESM in the population after MBS, we used an intensive sampling strategy over 14 consecutive days with 6 prompts per day, which is a strength of our study. Furthermore, we collected both quantitative and qualitative data to answer our primary research aim, which increased the validity of the study.

There are a few limitations to this study, so the results should be interpreted with caution. First, the relatively small sample size is a limitation of our study. However, the small sample size was justifiable, due to the large amount of collected data by intensive sampling, and to the exploratory nature of the study. Despite the small sample size, we add to the limited knowledge about the feasibility of ESM in the population after MBS. Second, we conducted multiple tests without correction, which may increase the risk of type I statistical errors, that is, finding significant results while there are no true underlying associations. In exploratory studies, the primary goal is to identify potential relationships, making it more appropriate to forego corrections. This approach allows for the identification of possible associations that can be further examined and validated in follow-up confirmatory studies. Third, the overall frequency of problematic eating behaviors in our study population was extremely low. To minimize recall bias, we asked participants about problematic eating behaviors occurring within the past 30 or 60 minutes, which may have missed behaviors occurring outside this time frame. Wider time windows could probably overcome this limitation, but this goes hand in hand with recall bias. Furthermore, behavior can change when monitored, even without an intended change, a phenomenon known as “measurement reactivity,” which may have increased participants’ awareness of their eating behaviors and led them to adjust their responses or actions accordingly. Together, this may have resulted in underreporting of problematic eating behaviors in our study. Finally, our assessment of binge eating focused solely on the quantity of food consumed, without evaluating LOC. Previous research highlights that LOC may be a stronger indicator of binge eating episodes, particularly in patients with obesity and patients undergoing MBS [58,59]. By focusing on quantity alone, we may have misclassified certain episodes or overlooked critical associations related to LOC. However, it is important to note that our primary aim was to assess the feasibility of ESM, which is not directly impacted by this limitation. For our secondary aim, the small number of binge eating episodes (n=7 across 3 participants) already posed challenges in detecting meaningful associations. Including the LOC aspect could have further reduced this number, potentially making statistical analyses even more difficult or infeasible. Nonetheless, we recognize that future studies should include LOC as a key criterion for binge eating to enhance content validity and better capture associations with momentary predictors.

### Future Perspectives

This study provides insights into the feasibility of ESM in the population after MBS. This information could be used to further refine the development of ESM to monitor patients during their postoperative trajectory. Several participants mentioned that the questionnaires were repetitive and monotonous. Compliance might be increased if the questions are asked in a different order. Furthermore, patients would appreciate a free-input text area to explain their answers about emotion and problematic eating behavior. Feasibility for health care personnel should be considered and evaluated in future studies, especially when the objective of ESM includes remote monitoring.

Monitoring efforts should be particularly directed toward individuals exhibiting problematic eating behaviors. Such results could help patients and health care professionals to identify problematic eating behavior episodes by recommending them to focus on specific emotions, physical states, or contextual factors.

### Conclusion

From this exploratory study to describe the feasibility of ESM regarding negative and positive affect, physical states, contextual factors, and problematic eating behaviors, findings suggest that ESM can be feasible in the population after MBS. Despite the low participation rate, compliance with experience sampling was moderate, and the completion rate was high. According to the interviews, patients considered ESM feasible and of added value, as awareness arose by using the ESM app. This offers a perspective for the use of ESM in the follow-up after MBS. Temporal associations were found between hunger and craving, and between positive affect and grazing. Nevertheless, no distinct patterns emerged among emotions, physical states, context, and problematic eating behaviors.

## Appendix

Multimedia Appendix 1 Semistructured interview guide.

Multimedia Appendix 2 Compliance in answering experience sampling questionnaires per participant during the 14-day study period.

Multimedia Appendix 3 Descriptive data for the different types of problematic eating behavior.

## Abbreviations

ESM: experience sampling methodology
GEE: generalized estimation equation
HUBBI: eHealth Usability Benchmarking Instrument
LOC: loss of control
MBS: metabolic bariatric surgery
OR: odds ratio
ZGT: Hospital Group Twente

## Data Availability

The data generated and analyzed during this study are not publicly available since participants were not asked for consent to make their data publicly available. The data are available from the corresponding author upon reasonable request.
