# Supplementary material for: Feasibility and Links Between Emotions, Physical States, and Eating Behavior in Patients After Metabolic Bariatric Surgery: Experience Sampling Study
Source: JMIR Form Res. 2025 Mar 5;9:e60486. doi: 10.2196/60486 (PMC11923469; doi:10.2196/60486)
Supplement: Multimedia Appendix 1 [file formative_v9i1e60486_app1.docx]

## Multimedia Appendix 1. Semistructured interview guide

**Themes to be discussed during the interview:**

- User-friendliness
- Technical performance
- Content and relevance
- Study design (questionnaire frequency, questions, duration)
- Positive and negative aspects
- Perceived advantages and disadvantages

**Questions (optional)**

1. What did you think of the application? What was your overall impression of the application?
2. What did you think of the usability of the application?
3. How did you experience answering the questionnaires?
4. What did you think of the study?
5. What did you think of the questions asked?
6. How did you feel about not having insight into your own data?
7. Suppose we are going to use the application in routine care after metabolic bariatric surgery. How do you think about that? How do you envision that?

If a participant quitted the study prematurely:

1. What was the reason you quitted the study before the end of the study? Could we have prevented you from quitting? How?
