# Supplementary figures and images for: Feasibility and Links Between Emotions, Physical States, and Eating Behavior in Patients After Metabolic Bariatric Surgery: Experience Sampling Study
Source: JMIR Form Res. 2025 Mar 5;9:e60486. doi: 10.2196/60486 (PMC11923469; doi:10.2196/60486)

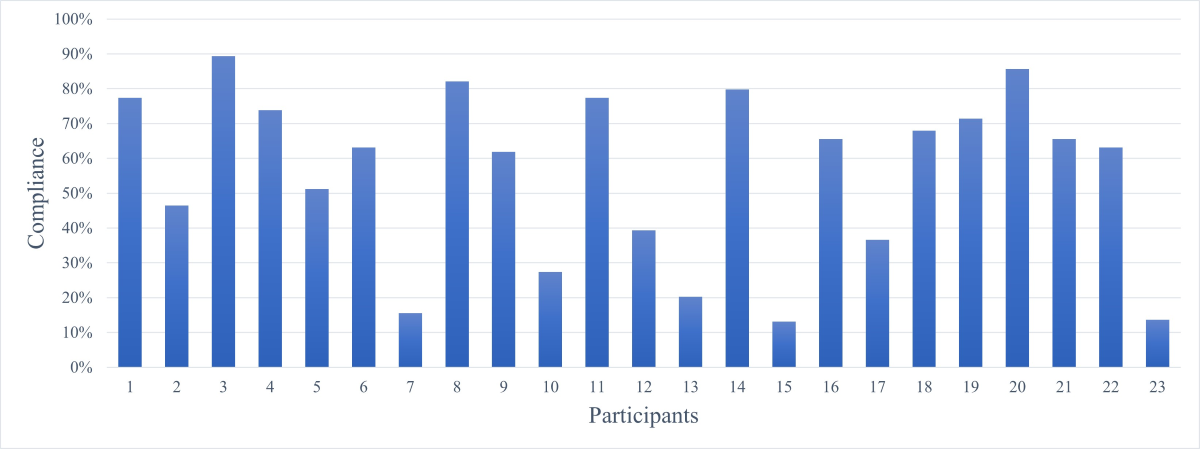

Supplement: Multimedia Appendix 2 [file formative_v9i1e60486_app2.png]
