# Supplementary material for: Feasibility and Links Between Emotions, Physical States, and Eating Behavior in Patients After Metabolic Bariatric Surgery: Experience Sampling Study
Source: JMIR Form Res. 2025 Mar 5;9:e60486. doi: 10.2196/60486 (PMC11923469; doi:10.2196/60486)
Supplement: Multimedia Appendix 3 [file formative_v9i1e60486_app3.docx]

## Multimedia Appendix 3: Descriptive data for the different types of problematic eating behavior

|  | | **Frequency** | **Percentage** |
| --- | --- | --- | --- |
| **Dietary relapse** | |  |  |
|  | Yes | 45 | 3 |
|  | No | 947 | 63 |
|  | Missing | 518 | 34 |
| **Grazing** | |  |  |
|  | Yes | 13 | 1 |
|  | No | 974 | 65 |
|  | Missing | 523 | 35 |
| **Binge eating** | |  |  |
|  | Yes | 7 | 1 |
|  | No | 983 | 65 |
|  | Missing | 520 | 34 |
| **Craving** | |  |  |
|  | 0 | 643 | 43 |
|  | 1 | 228 | 15 |
|  | 2 | 54 | 4 |
|  | 3 | 24 | 2 |
|  | 4 | 11 | 1 |
|  | 5 | 12 | 1 |
|  | 6 | 10 | 1 |
|  | 7 | 4 | 0 |
|  | 8 | 7 | 1 |
|  | 9 | 1 | 0 |
|  | 10 | 0 | 0 |
|  | Missing | 516 | 34 |
